# Supplementary material for: Nanoscale imaging of pT217‐tau in aged rhesus macaque entorhinal and dorsolateral prefrontal cortex: Evidence of interneuronal trafficking and early‐stage neurodegeneration
Source: Alzheimers Dement. 2024 Mar 6;20(4):2843–60. doi: 10.1002/alz.13737 (PMC11032534; doi:10.1002/alz.13737)
Supplement: Supplementary file 1 — Supporting Information [file ALZ-20-2843-s001.docx]

**Title:** Nanoscale imaging of pT217-tau in aged rhesus macaque entorhinal and dorsolateral prefrontal cortex: Evidence of interneuronal trafficking and early-stage neurodegeneration

**Authors:** Dibyadeep Datta^1,2^*, Isabella Perone^1^, Denethi Wijegunawardana^1^, Feng Liang^3^, Yury M. Morozov^1^, Jon Arellano^1^, Alvaro Duque^1^, Zhongcong Xie^3^, Christopher H. van Dyck^2^, Mary Kate P. Joyce^1^, Amy F.T. Arnsten^1^*

**Affiliations:**

^1^Departments of Neuroscience, Yale University, School of Medicine, 333 Cedar St., New Haven, CT USA 06510

^2^ Department of Psychiatry, Yale University, School of Medicine, 333 Cedar St., New Haven, CT USA 06510

^3^ Department of Anesthesia, Critical Care and Pain Medicine, Massachusetts General Hospital and Harvard Medical School, 55 Fruit Street, Boston, MA 02114

* Corresponding Authors

**Corresponding authors:**

Dr. Amy F.T. Arnsten, Ph.D.,

Albert E. Kent Professor of Neuroscience

Dept. Neuroscience, Yale Medical School

333 Cedar St., New Haven, CT USA 06510

Phone: 203-785-4431

Fax: 203-785-5263

Email: [amy.arnsten@yale.edu](mailto:amy.arnsten@yale.edu)

Dibyadeep Datta, Ph.D.

Assistant Professor

Department of Psychiatry

Yale School of Medicine

333 Cedar St.

New Haven CT 06510

Phone: 203-785-5283

Email: [dibyadeep.datta@yale.edu](mailto:dibyadeep.datta@yale.edu)


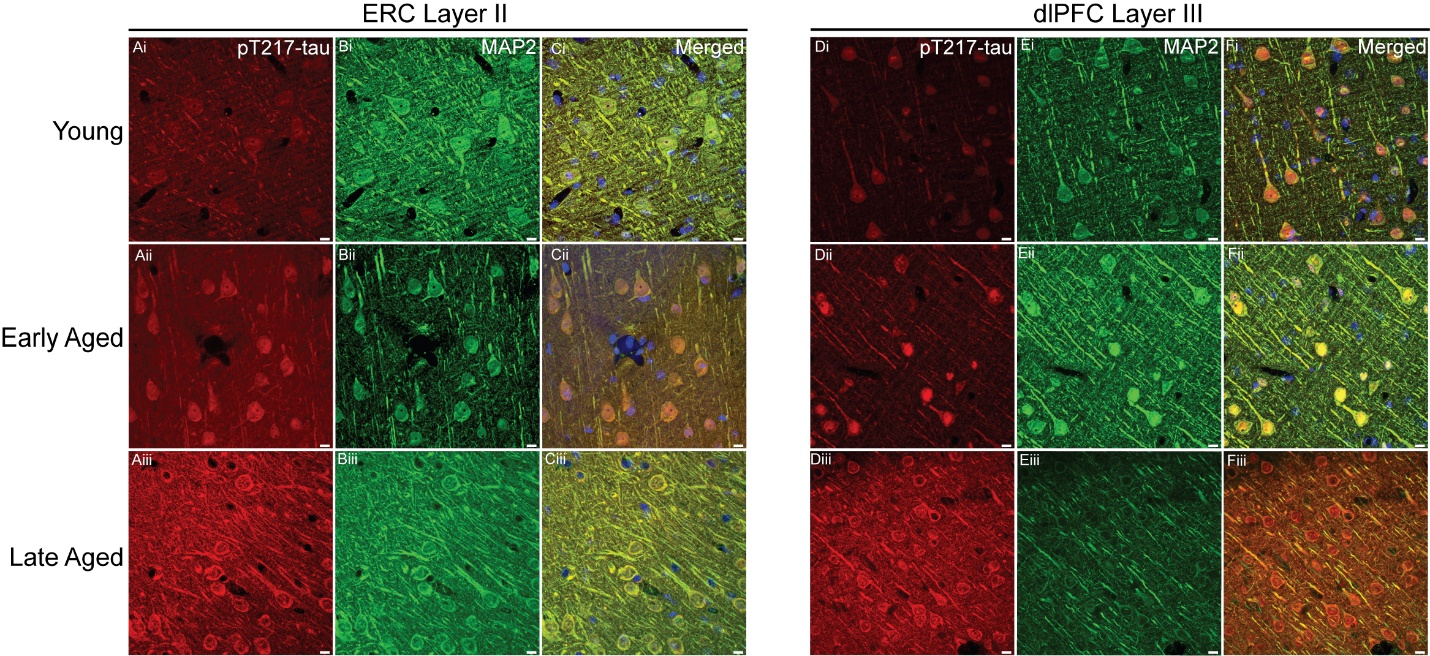


**Supplementary Figure 1. Spatial and temporal localization pattern of pT217-tau across vulnerable cortical regions.** Multiple-label immunofluorescence showing pT217-tau labeling (red) co-localized in excitatory neurons (MAP2, green) in ERC layer II and dlPFC layer III across age-span in rhesus macaques at low-magnification. In adult (8-10 years) rhesus macaques, pT217-tau immunolabeling was observed within the perisomatic compartment and the nucleus in a subset of excitatory cells **(Ai-Ci, Di-Fi)**, including delicate expression in proximal apical dendrites in ERC layer II cell islands and dlPFC layer III microcircuits. With advancing age, immunofluorescence revealed pT217-tau accumulating in proximal and more distal apical and basal dendrites in ERC layer II excitatory cells and dlPFC deep layer III pyramidal cells, both in “early” aged monkeys (18-19 y; **Aii-Cii, Dii-Fii**) and “late” aged monkeys (28-34y; **Aiii-Ciii, Diii-Fiii**). Note that some of what appears as possible “background labeling” in light microscopy likely includes neuropil labeling, based on the extensive pT217-tau labeling in dendritic spines and distal dendrites seen with immunoEM. Scale bars: 10μm.

**
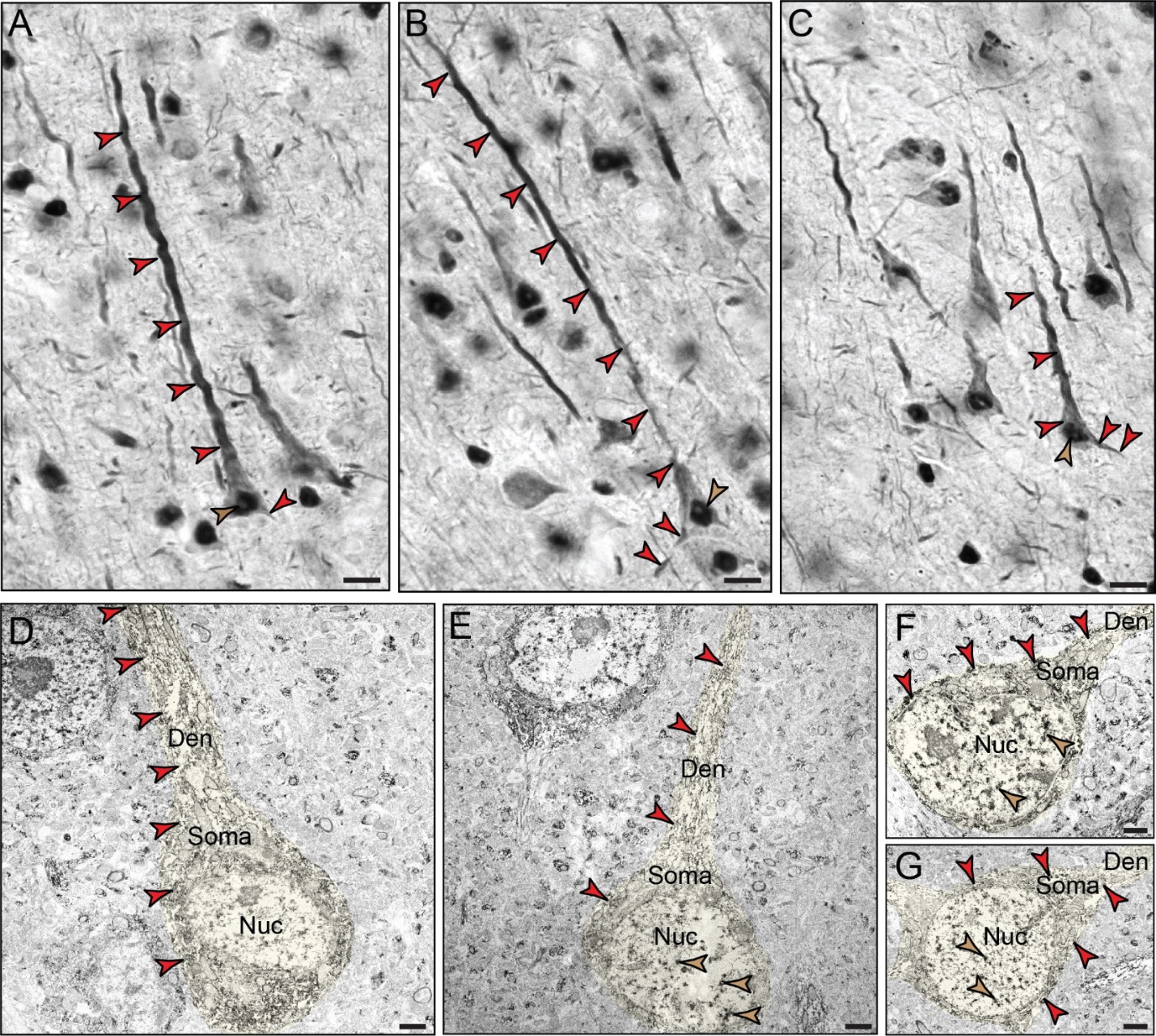
**

**Supplementary Figure S2. Immunolabeling for pT217-tau in aging rhesus macaques.** High-magnification brightfield micrographs revealing intense pT217-tau immunoreactivity along apical and basal pyramidal dendrites (red *arrowheads*), cell soma, and nucleus in aged (30-31 y) rhesus macaque dlPFC layer III **(A-C)**. pT217-tau immunolabeling of pyramidal cells showed aggregated, filamentous pattern within apical dendrites, often with a twisted morphology. Diffuse immunoreactivity is observed in the neuropil, including punctate labeling. Scale bars: 10µm. Low magnification immunoEM micrograph showing pT217-tau immunoperoxidase immunolabeling in pyramidal neurons with a triangular shaped cell body in dlPFC deep layer III **(D-G)**. The pT217-tau immunolabeling is observed in the cell soma and extending along the apical and basilar dendrite (pseudocolored in yellow). Immunolabeling for pT217-tau is also observed in a subset of neurons in the nucleus (indicated by brown *arrowheads*), correlating with light-level immunoperoxidase immunolabeling. pT217-tau protein is also observed in the neuropil. Scale bar: 2µm.

**
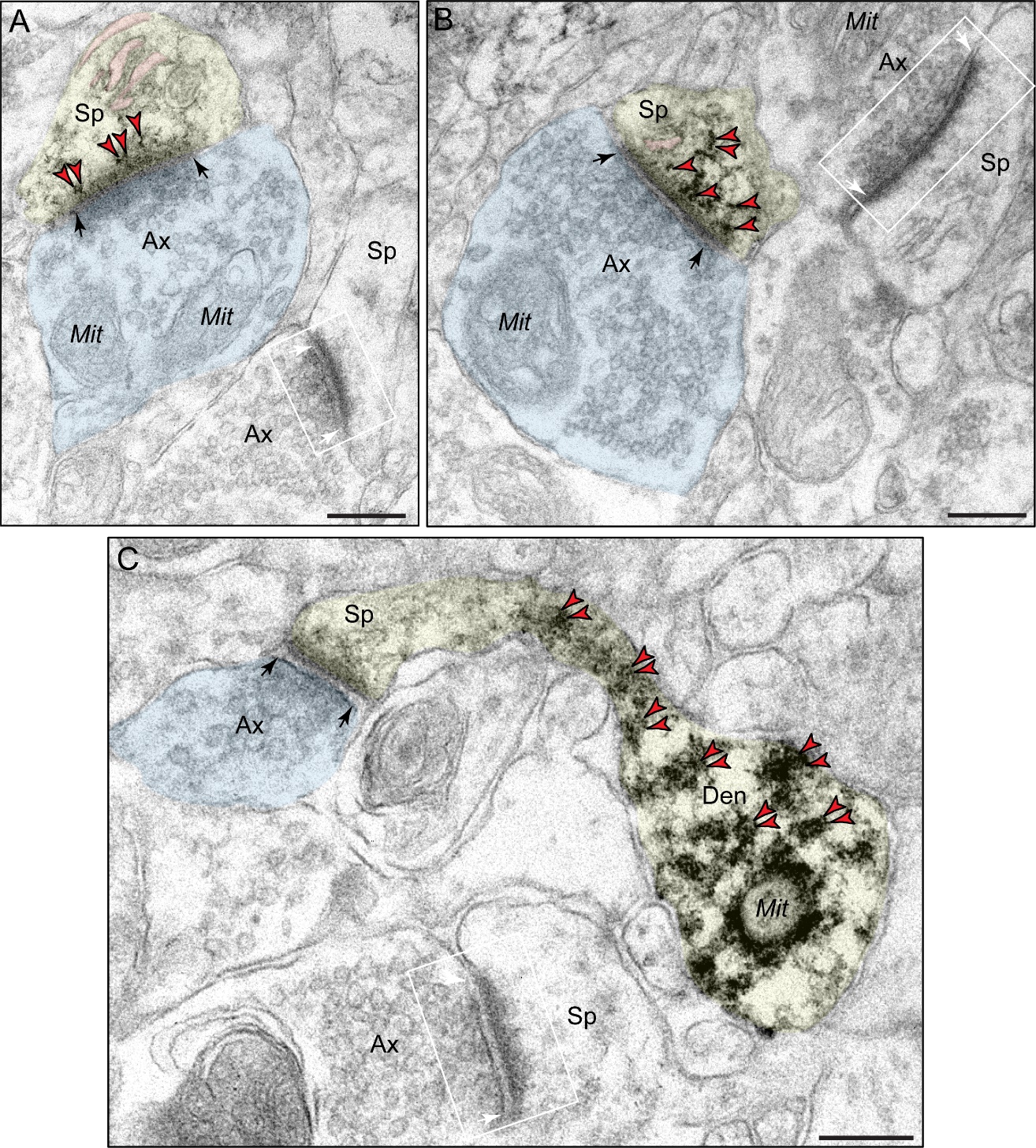
**

**Supplementary Figure S3. Contrasting immunopositive and immunonegative pT217-tau elements with immunoEM.** A comparison of immunopositive vs. immunonegative (white rectangles) synapses to help distinguish between the electron dense post-synaptic density seen in all synapses, and the darker immunoperoxidase label. As detailed in the main text, the immunoperoxidase labeling revealed that pT217-tau immunolabeling was concentrated in dendritic spines near asymmetric, glutamate-like synapses, and in dendrites in “late” aged (26-31y) macaque dlPFC layer III **(A-C)**. An example of delicate expression of pT217-tau can be seen in **(A)**, allowing visualization of synaptic details, where labeling was observed in association with omega-shaped endosome-like vesicular profiles on the plasma membrane within dendritic spines, specifically within the postsynaptic density, near axospinous, asymmetric glutamatergic synapses. pT217-tau is also observed directly in association with the smooth endoplasmic reticulum (SER) spine apparatus (pseudocolored pink) **(B)**. In a rare capture in **(C)**, pT217-tau immunolabeling is observed in the dendritic spine neck, emanating from a dendritic shaft that is also immunopositive, where pT217-tau immunolabeling can be visualized aggregating on microtubules. Synapses are between arrows. Red *arrowheads* point to examples of pT217-tau immunoreactivity. In contrast, pT217-tau immunonegative dendritic spines are highlighted in the neuropil in the same micrographs **(A-C)**. In these examples, the synapse is highlighted (white arrows with white rectangle) to show lack of pT217-tau immunoreactivity, confirming the specificity of the immunolabeling method. The dendrites and dendritic spines are pseudocolored yellow, and the axon terminals are pseudocolored blue, to enhance clarity. Ax, axon; Sp, dendritic spine; Mit, mitochondria. Scale bars, 200 nm.

**
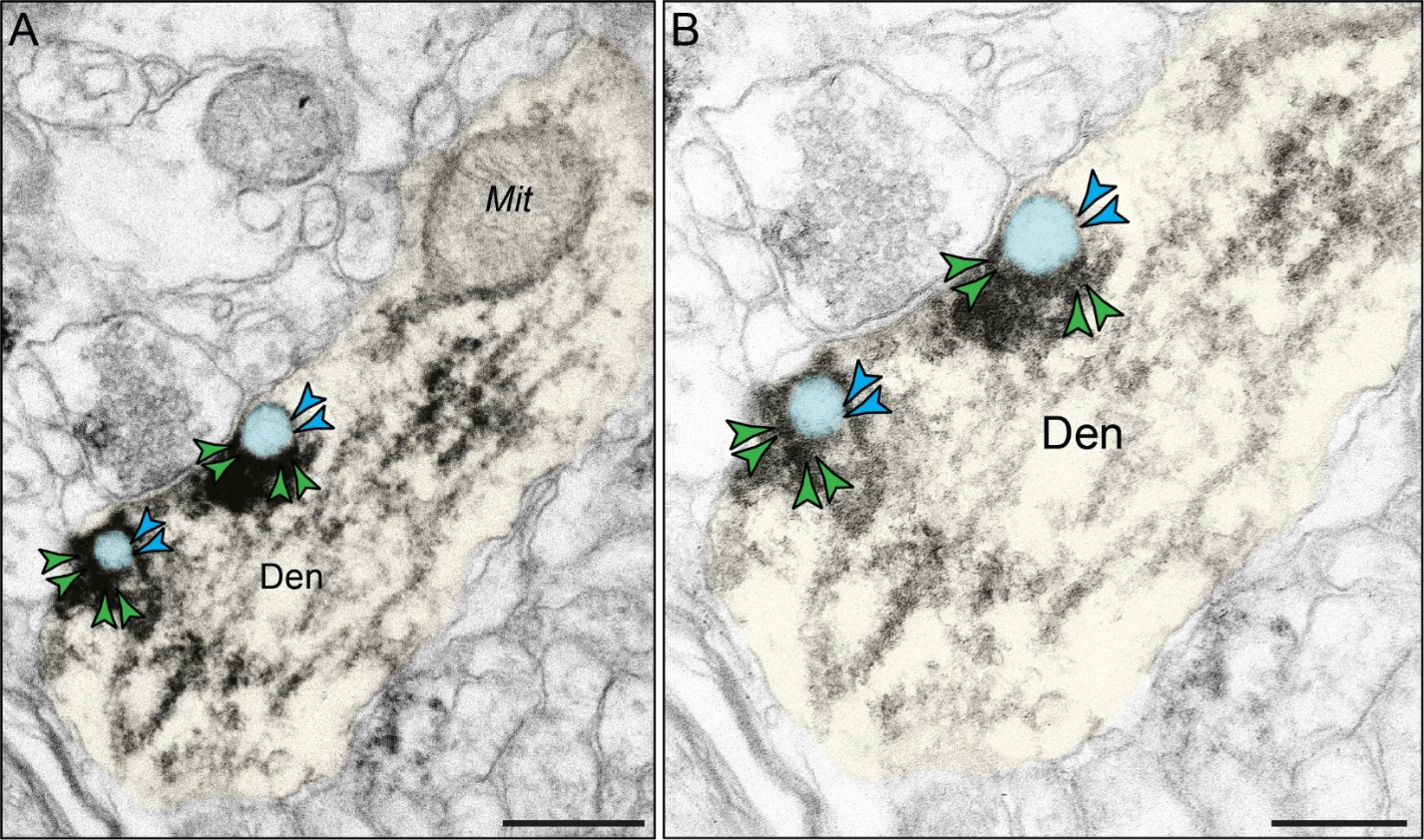
**

**Supplementary Figure S4. Immunolabeling for endosomes by the marker EEA1 in aged rhesus macaque.** ImmunoEM characterization of endosomes (pseudocolored in cyan and cyan *arrowheads*) in aged macaque (25 years) dlPFC layer III using EEA1 (green *arrowheads*), a canonical marker of early endosomes. EEA1 selectively labels endosomes at the ultrastructural level, revealing membrane-bound organelles with a translucent cytosol. Endosomes in aged rhesus macaque appear morphologically enlarged within dendritic shafts near microtubule bundles and mitochondria in aged macaque (25 years) dlPFC layer III **(A)**. Higher-magnification micrograph showing selective labeling of EEA1 with endosomes within dendritic shafts in aged macaque (25 years) dlPFC layer III **(B)**. The dendrites are pseudocolored yellow for clarity. Den, dendrite; Mit, mitochondria. Scale bars, 200 nm (A), 100 nm (B).


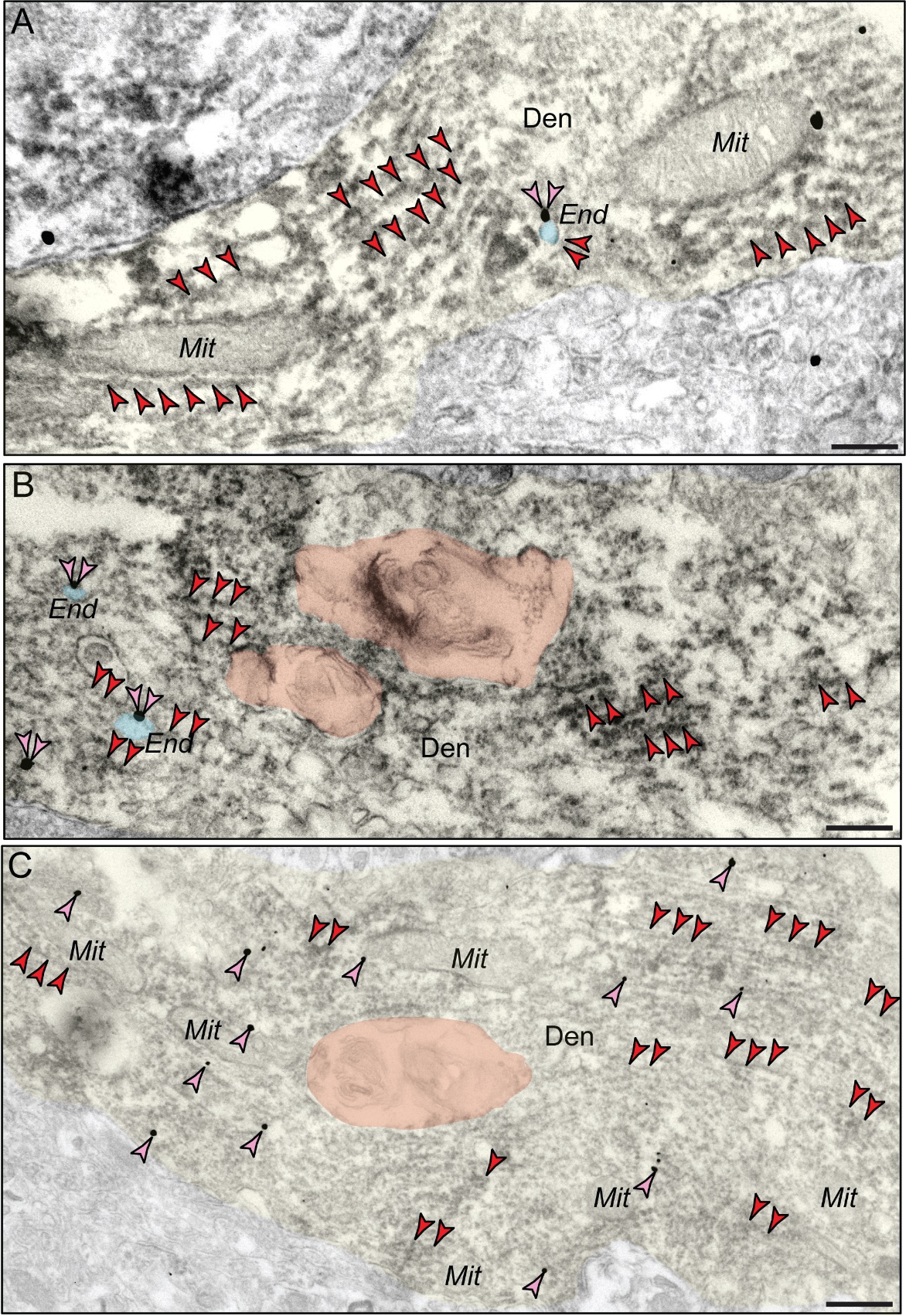


**Supplementary Figure 5**. **Evidence of pT217-tau surrounding endosomes associated with Aβ42 in dendritic shafts, with concordant signatures of autophagic degeneration.**

Using dual-label immunoEM, the current data show evidence of pT217-tau (DAB labeling; indicated by red *arrowheads*) surrounding endosomes (pseudocolored in cyan) containing Aβ42 (immunogold labeling; indicated by pink *arrowheads*) in dendrites in aged macaque dlPFC, suggesting that pT217-tau may be involved in the etiology of amyloid pathology (**Supplementary Figure 5A**). We also observe extensive evidence of pT217-tau and Aβ42 co-localized within dendrites with concordant signatures of autophagic vacuolar degeneration with multilamellar bodies (pseudocolored in orange) (**Supplementary Figure 5B-C**). The dendrites are pseudocolored yellow for clarity. End, endosome; Den, dendrite; Mit, mitochondria. Scale bars, 200 nm.


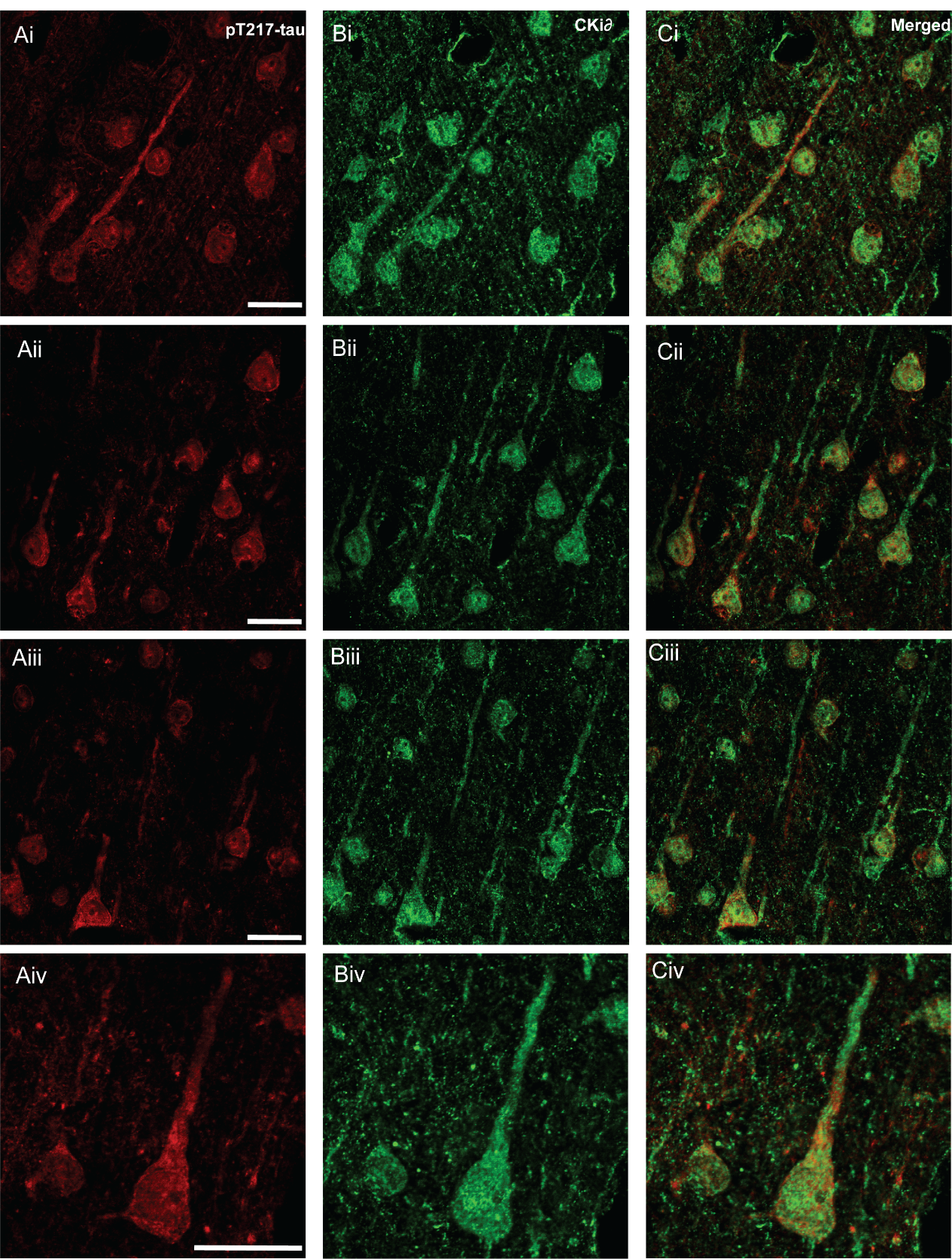


**Supplementary Figure 6. Co-localization between of pT217-tau and CKI∂, a marker of granulovacuolar degeneration bodies, in late aged rhesus macaque dlPFC.** Multiple-label immunofluorescence showing pT217-tau labeling (red) co-localized in excitatory neurons with CKI∂ (green) in dlPFC layer III in a “late” aged rhesus macaque (30 y). A punctate pattern of co-localization can be seen throughout the apical dendrite and in the perisomatic subcompartment of pyramidal cells. Scale bars: 24μm.


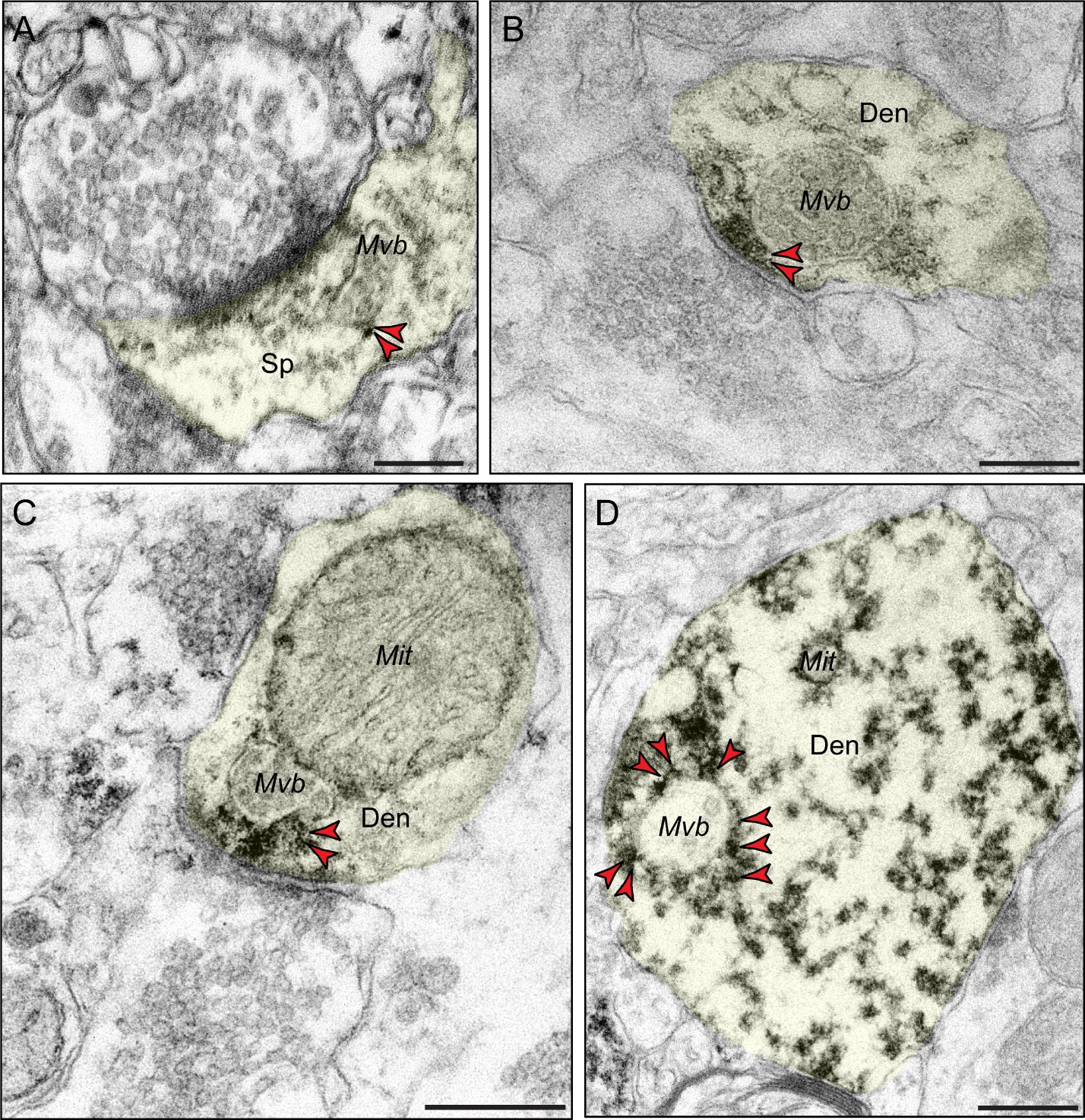


**Supplementary Figure 7. Association of pT217-tau with multivesicular bodies in rhesus macaque ERC and dlPFC.** pT217-tau (indicated by red *arrowheads*) associated with multivesicular bodies in dendritic spines and dendritic shafts in “early” aged (18-24y) macaque ERC layer II and in “late” aged (26-31y) macaque dlPFC layer III **(A-D)**. These results corroborate recent studies that have shown that a strong relationship between pT217-tau and multivesicular bodies in postmortem human AD brain (Wennstrom et al. *Acta Neuropathological Communications* 2022). Profiles are pseudocolored for clarity. Sp, dendritic spine; Den, dendrite; Mit, mitochondria. Scale bars, 200 nm.


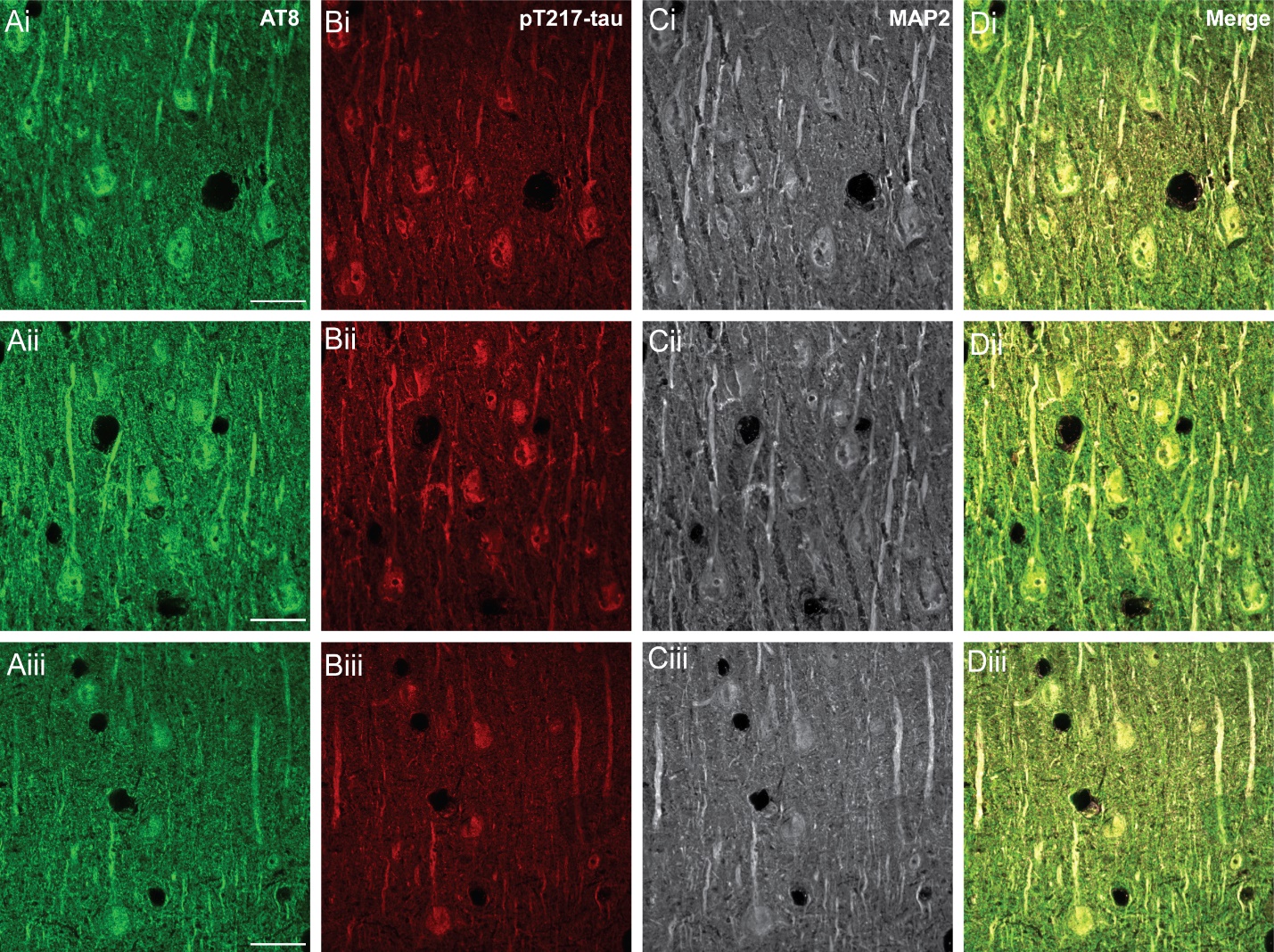


**Supplementary Figure 8. Multi-label immunofluorescence for pT217-tau and pSer202/pThr205-tau (AT8) in late aged rhesus macaque dlPFC.** Multiple-label immunofluorescence showing pT217-tau labeling (red) co-localized in pyramidal neurons with AT8 (green), and MAP2 (white) in dlPFC layer III in a “late” aged rhesus macaque (30 y). Co-localization of pT217-tau and AT8 co-localization is prominently observed along the apical dendrites of layer III pyramidal cells. Scale bars: 24μm.
